# Concurrent TMS-fMRI: Technical Challenges, Developments, and Overview of Previous Studies

**DOI:** 10.3389/fpsyt.2022.825205

**Published:** 2022-04-21

**Authors:** Yuki Mizutani-Tiebel, Martin Tik, Kai-Yen Chang, Frank Padberg, Aldo Soldini, Zane Wilkinson, Cui Ci Voon, Lucia Bulubas, Christian Windischberger, Daniel Keeser

**Affiliations:** ^1^Department of Psychiatry and Psychotherapy, University Hospital LMU, Munich, Germany; ^2^Neuroimaging Core Unit Munich - NICUM, University Hospital LMU, Munich, Germany; ^3^High Field MR Center, Center for Medical Physics and Biomedical Engineering, Medical University of Vienna, Vienna, Austria; ^4^International Max Planck Research School for Translational Psychiatry, Munich, Germany; ^5^Department of Radiology, University Hospital LMU, Munich, Germany

**Keywords:** concurrent TMS-fMRI, interleaved TMS-fMRI, functional MRI (fMRI), transcranial magnetic stimulation (TMS), review

## Abstract

Transcranial magnetic stimulation (TMS) is a promising treatment modality for psychiatric and neurological disorders. Repetitive TMS (rTMS) is widely used for the treatment of psychiatric and neurological diseases, such as depression, motor stroke, and neuropathic pain. However, the underlying mechanisms of rTMS-mediated neuronal modulation are not fully understood. In this respect, concurrent or simultaneous TMS-fMRI, in which TMS is applied during functional magnetic resonance imaging (fMRI), is a viable tool to gain insights, as it enables an investigation of the immediate effects of TMS. Concurrent application of TMS during neuroimaging usually causes severe artifacts due to magnetic field inhomogeneities induced by TMS. However, by carefully interleaving the TMS pulses with MR signal acquisition in the way that these are far enough apart, we can avoid any image distortions. While the very first feasibility studies date back to the 1990s, recent developments in coil hardware and acquisition techniques have boosted the number of TMS-fMRI applications. As such, a concurrent application requires expertise in both TMS and MRI mechanisms and sequencing, and the hurdle of initial technical set up and maintenance remains high. This review gives a comprehensive overview of concurrent TMS-fMRI techniques by collecting (1) basic information, (2) technical challenges and developments, (3) an overview of findings reported so far using concurrent TMS-fMRI, and (4) current limitations and our suggestions for improvement. By sharing this review, we hope to attract the interest of researchers from various backgrounds and create an educational knowledge base.

## Basic Information

### Why Should TMS Be Combined With FMRI?

Transcranial magnetic stimulation (TMS) is a non-invasive transcranial brain stimulation (NIBS) technique, which modulates neuronal activity by applying electromagnetic pulses to the scalp. A unique strength of TMS is that it allows for an experimental *in-vivo* investigation by depolarizing neurons to induce action potentials. TMS can be applied either as single-pulse TMS (sTMS) or repetitive TMS (rTMS). sTMS is often used to investigate the functional role of a particular region by interfering or otherwise modulating specific cortical activities. Various rTMS protocols can be administered; for example, low-frequency and high-frequency rTMS, which are commonly used to induce inhibitory or facilitatory effects, respectively (1). rTMS is frequently used as a treatment for neurological and psychiatric patients (2). The most recent guideline for the therapeutic usage of rTMS shows level A evidence for depression, motor stroke, and neuropathic pain, as well as level B evidence in fibromyalgia, Parkinson's disease, multiple sclerosis, and post-traumatic stress disorder (2). The U.S. Food and Drug Administration (FDA) has approved rTMS as a therapeutic intervention for pharmacotherapy-non-responsive major depressive disorder (MDD) (3–5), obsessive-compulsive disorder (OCD) (6), smoking cessation (7), and migraine (8). Recently, intermittent theta-burst stimulation (iTBS), an rTMS variant, has also been approved by the FDA for depression therapy. Theta-burst stimulation (TBS) replicates a typical firing pattern of hippocampal neurons during the learning process. Among the TBS protocols, iTBS is a very short (e.g., 3 min) and safe protocol that mimics hippocampal TBS patterns to induce long-term potentiation (LTP) in basic neurophysiology (9, 10), and exerts, presumably at least, equally robust post-stimulation effects compared to longer standard protocols (3, 11).

Although rTMS is already used in therapy, the mechanism of TMS-mediated neuronal modulation is not yet fully understood. Over the last decades, various neuroimaging techniques have been combined with TMS to investigate neuronal activation changes due to stimulation: electroencephalography (EEG), near-infrared spectroscopy (NIRS), positron emission tomography (PET), and functional magnetic resonance imaging (fMRI) (12, 13). However, despite the importance of fMRI for mapping neuronal activity, the majority of the studies to date involve concurrent TMS-EEG (14, 15). Nonetheless, compared to EEG, fMRI boasts superior spatial resolution, especially when functional connectivity is of interest. A combination of TMS and fMRI can provide us with meaningful information that helps us elucidate the underlying mechanisms of TMS-mediated neuronal modulation.

### How Can TMS Be Concurrently Applied With FMRI?

As fMRI data quality heavily depends on magnetic field homogeneity, concurrent application of strong TMS pulses during imaging would cause severe artifacts. Hence, TMS is often applied offline, i.e., separately from the actual imaging procedure, and cerebral activation after the TMS session is compared to a pre-TMS baseline. Obviously, such approaches are limited in their sensitivity for capturing post-stimulation effects, and immediate stimulation effects are likely to be undetected.

**Figure 1:**
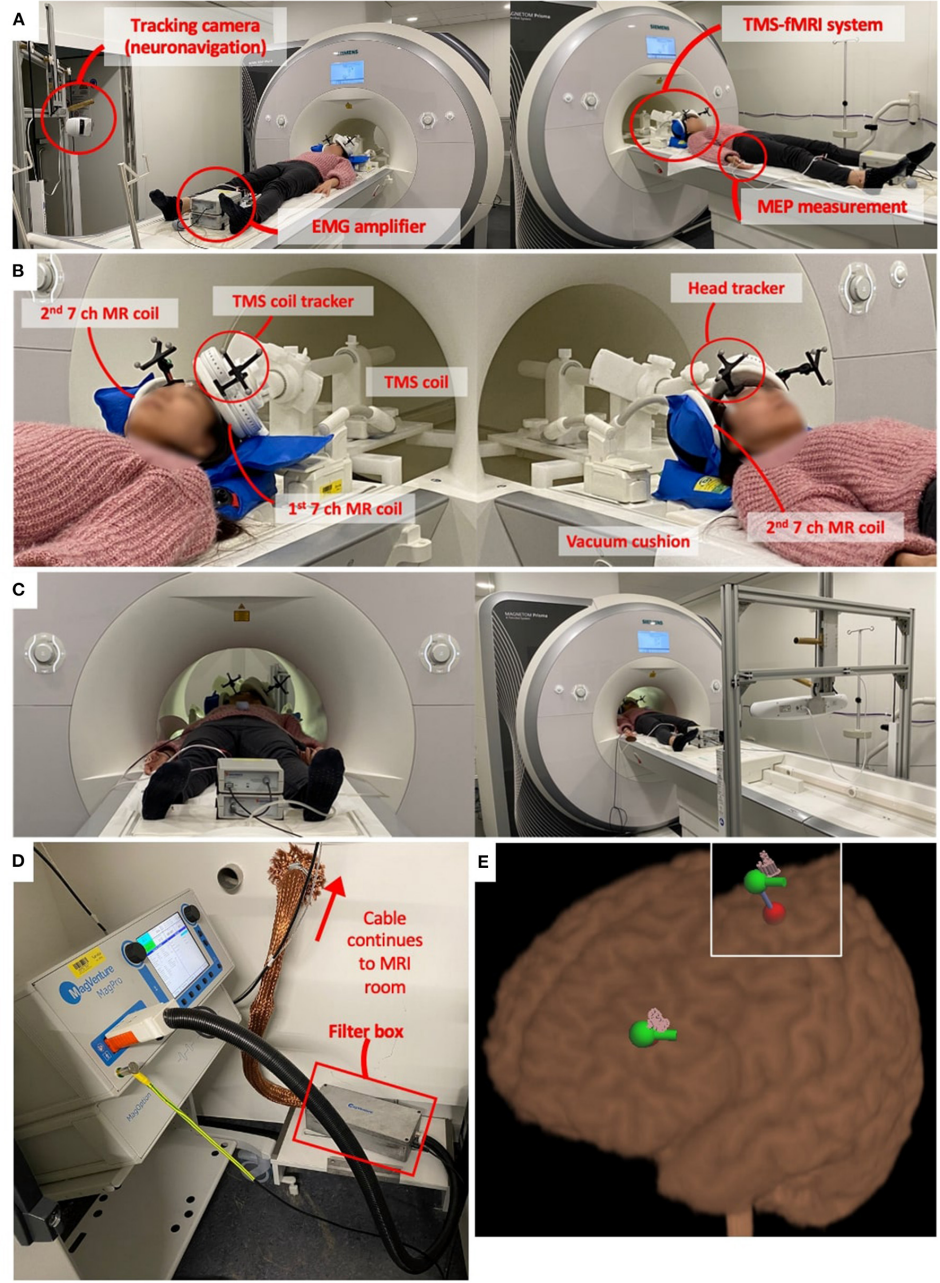
Example of TMS-fMRI set up. **(A)** This particular example of TMS-fMRI set up includes MR compatible TMS coils (Magventure, Farum, Denmark), MR coils (16), neuronavigation system (Localite, Bonn, Germany), and a BrainAmp ExG MR amplifier (BrainProducts, Gilching, Germany) for electromyographic (EMG) measurements to determine motor threshold (MT). **(B)** TMS coil is mounted on a holder which goes in the bore of the scanner. The holder system is attached to the scanner bed so that it moves together. The MR head coil, in this example, is two thin and flat seven channel coils. One is attached below the TMS coil, and the other is stabilized on the other side of the head using a vacuum pillow. Two trackers on the forehead and coil enable neuronavigation. **(C)** EMG amplifier continues to record the motor evoked potential (MEP) and the neuronavigation system tracks the TMS coil location throughout the scanning session. **(D)** TMS device remains in the technical room as it is ferromagnetic. The TMS coil is connected to the MRI room by a 6-m cable through a hole in the wall. The cable is covered with a filter tube, and a filter box is installed along the cable. **(E)** Neuronavigation system shows the location of the stimulation. The red dot is calculated with a coordinate that defines the target point. By defining the coil orientation, it calculates the entering point which is the green dot with the green bar showing the coil handle orientation. The pink pins show the actual stimulation location, which is recorded each time a TMS pulse is applied during the TMS-fMRI session. These pink pins can be recorded during the concurrent TMS-fMRI session and can be used for the post-analysis as far as the head and coil trackers are visible in the MR bore as shown in panel **(C)**.

To address this problem, MR-compatible TMS systems have been developed which enable online TMS experiments, where TMS is applied inside the MR-scanner during image acquisition. Figure 1 shows an example of such a TMS-fMRI setting. The TMS stimulator typically stays outside the MR room. A long cable through the wall connects the TMS main unit with the TMS coil in the scanner bore. The MR head coil can be set up in various ways (discussed in section MR Head Coil and TMS Positioning System), but in this specific example, the thin MR coil is attached to the TMS coil. This TMS-fMRI setting allows subjects to remain in the MR bore for the entire duration of the MRI scan acquisition while receiving TMS inside the scanner bore.

**Figure 2:**
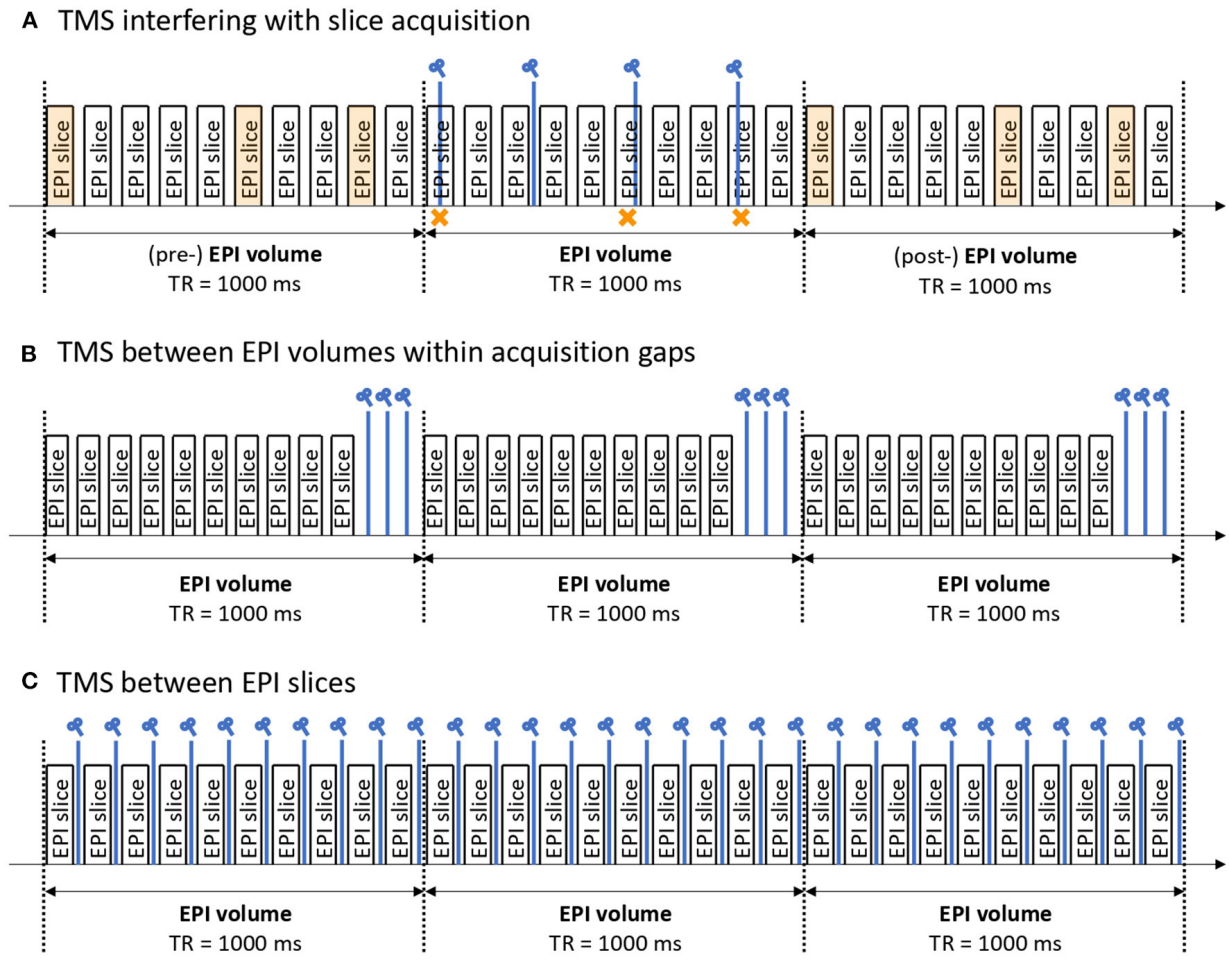
Example of concurrent TMS-fMRI protocols. There are three possible ways to apply TMS pulses during fMRI simultaneously. **(A)** A method enabling TMS to interfere with EPI slices. Perturbed EPI slices (indicated by an orange cross) are sacrificed and replaced by slice interpolation, typically from the volumes before and after the volume of interest (indicated by orange EPI slices). Advantages: High flexibility to stimulate at any time regardless of EPI timing. Disadvantages: It requires a high level of post-processing capabilities to detect the damaged EPI slices and replace them. **(B)** A method to insert a gap time between EPI slices and apply TMS pulses meanwhile. Advantages: No EPI slices are sacrificed. Disadvantages: The number of stimulations is limited as it needs to fit in the gap time. Hence, the whole TMS-fMRI protocol tends to be longer. **(C)** A method to interleave TMS pulses with EPI slices. Advantages: It allows for continuous stimulation which is often used as a therapeutic protocol. Disadvantages: Reliable hardware and software are essential as the pulses must be controlled precisely.

Furthermore, careful interleaving of TMS pulses with MR signal acquisition enables continuous fMRI scanning. In the field of TMS research, this technique is referred to as either concurrent or simultaneous TMS-fMRI with off-/online block or interleaved designs. The feasibility of the concurrent TMS-fMRI method was first demonstrated in 1998 (17) when TMS pulses were successfully applied during the gap time between slice acquisitions. Currently, there are three possible methods of concurrent TMS-fMRI approach (Figure 2).

Figure 2A illustrates how to interfere with echo-planar imaging (EPI) slices using TMS. Perturbed EPI slices (indicated by orange crosses in Figure 2A) are sacrificed and replaced by interpolation of slices, typically from the volumes before and after the volume of interest (indicated by orange EPI slices in Figure 2A). The advantage of this method is the high flexibility for stimulating at any time regardless of EPI timing, whereas the disadvantage is that it requires a high level of post-processing capabilities to detect the damaged EPI slices and replace them.

Figure 2B illustrates how to insert a gap time between EPI slices and then apply TMS pulses during this time. The advantage of this method is that no EPI slices are sacrificed, whereas the disadvantage is that the number of stimulations is limited as it needs to fit within the gap time. By virtue of these gap times, the whole TMS-fMRI protocol tends to become longer.

Figure 2C illustrates how to interleave TMS pulses with EPI slices. The advantage of this method is that it allows for continuous stimulation, which is often used as a therapeutic protocol. On the other hand, the disadvantage is that reliable hardware and software are essential, as the pulses must be controlled precisely.

## Technical Considerations

One of the most critical considerations in concurrent TMS-fMRI experiments concerns the TMS coil to be used in the MR scanner, particularly the materials used in its manufacturing. This is due to two reasons: (1) to guarantee the safety of the subjects by avoiding any attraction forces of ferromagnetic material in the magnetic field; (2) to withstand Lorentz forces during the stimulation within the high magnetic field inside the MR scanner. Therefore, coil composition is suggested to include a copper coil and a robust plastic housing to make the coil safe, durable in the magnetic field, and affordable (18, 19).

A further challenge with combining TMS and fMRI is to avoid artifacts in the image induced by TMS. The source of artifacts and noise caused by TMS can be categorized into three domains: (1) magnetic field, (2) radio frequency (RF) noise, and (3) leakage currents. These factors are critical for intra-individual test-retest reliability of fMRI data in response to TMS (20). In the following, appropriate technical suggestions and developments are discussed.

### Controlling for Magnetic Field Interference

Data quality in functional MRI depends on a high homogeneity of the static magnetic field. As TMS coils are heavy metallic objects, field homogeneity is compromised by the TMS coil via susceptibility-related field changes. Field inhomogeneities lead not only to signal losses, via intra-voxel dephasing effects, but also to geometric distortions in the images acquired. Several recommendations have been made to control for these magnetic field interferences.

A straightforward approach is to increase the distance between the head and TMS coil. However, the effect of TMS measured by motor threshold (MT) is known to decline as a function of the coil-cortex distance (21, 22). Baudewig et al. (23) showed severe signal loss and geometric distortions when TMS-fMRI scans are performed on test objects, while no such artifacts were seen in subject scans. They concluded that the distance between the head and cortical surface (typically 15–25 mm) is sufficient to minimize such artifacts. Hence, it is important to consider the optimal coil-cortex distance to preserve the TMS effect while minimizing artifacts.

Another essential factor to consider is the timing of the TMS pulse relative to fMRI data acquisition. Bestmann et al. (24) suggested that a period of at least 100 milliseconds (ms) before EPI onset is sufficient. However, the actual timing is a matter of the sequence and hardware used, and 100 ms spacing is not always required. Recently, due to newly developed protocols and the possibility of more precise timing, a shorter acquisition gap is allowed which improves the scanning quality. The important factors which could lead to a shorter delay include the sequence trigger configuration, stimulator model, software version, and any control units between scanner and stimulator, such as scripts used to control the stimulator based on received scanner triggers. Nevertheless, the most important factor for mitigating EPI distortions is the optimal delay length, which depends on various factors. Therefore, there is a degree of trial-and-error in the beginning when establishing the interleaved protocol for each TMS-fMRI setup.

It is also important to consider that the fMRI setting should suit the protocols employed. Baudewig et al. (23) suggested to keep the plane of the TMS coil parallel to the EPI section for a better signal-to-noise ratio (SNR). Bestmann et al. (24) recommended to keep it parallel to the frequency-encoding gradient. These suggestions from previous studies should be considered when planning the fMRI sequences. However, it is worth noting that these suggestions are not always feasible. For example, when the study protocol includes dorsolateral prefrontal cortex (DLPFC) stimulation and whole brain imaging, the above recommendations might not be advantageous. In reality, they may necessitate large volumes and significantly increase acquisition time. It is important to try different phase/frequency encoding directions, as well as EPI orientation, to see which configuration results in the best SNR and least artifacts at the same time.

### Controlling Radio Frequency Noise

Noise signals from the control room can penetrate the scanner room via TMS coil cables. These coil cables can act as transmitting antennas for RF noise which causes artifacts and decreases the SNR (25). In principle, the best method to avoid outside RF noise is to locate the TMS stimulator within the MR scanner's Faraday cage, fit the TMS cable in ferrite cable traps, and channel the cable through an RF filter box (26–28). However, these approaches have potential danger due to the attractive forces of the scanner field on the stimulator and cable traps and should thus be avoided, particularly when high field systems (3T and above) are used (29). Instead, Bungert et al. (29) reported that using in-line RF-filters is sufficient for avoiding artifacts (only 3% SNR loss), though such a filter reduces the functional efficacy of TMS by around 7%. In recent TMS-fMRI settings, it is common to filter all the cables which are entering the MRI room to avoid RF noise propagating.

### Controlling Leakage Currents

A TMS stimulator typically uses high-voltage capacitors that generate strong currents. These strong currents are delivered through the TMS coil when capacitors are discharged. However, small residual currents leak to the TMS coil even when the TMS coil is not discharged. These small leakage currents can cause magnetic field inhomogeneities resulting in spatial distortions in the EPI (30). Leakage currents need particular attention in TMS-fMRI experiments where TMS intensity is systematically varied, as this causes changes in the level of capacitor charges (31). To avoid these artifacts, Weiskopf et al. (31) suggested inserting a relay with minimal resistance in parallel, as well as two high-voltage diodes in series with the TMS coil. This relay-diode combination allows leakage current to flow through the relay and not through the TMS coil, thereby reducing artifacts considerably. Most current installations use a similar approach where a filter box is mounted at the filter plate. It includes a relay that redirects the leaking currents through the shorting box while the TMS coil is inactive, which mimics the effect of the relay-diode combination.

### MR Head Coil and TMS Positioning System

The choice of MR coil and TMS holder are fundamental to the preparation of concurrent TMS-fMRI experiments. Regarding the MR coil, several approaches have been used including 12-channel head coils (32, 33), 8-channel head coils (34–36), 6-channel flexible coils (37, 38), FLEX-L coils (2 circular RF receive coils) (39, 40), standard circular-polarized (CP) head coils (26, 31, 41–43) and simple surface coils (28, 44, 45). Typically, excitation is accomplished with the standard transmitter body coil. In all these setups, the TMS coil is placed inside the RF coil. With birdcage coils, the flexibility of the TMS coil localization is limited. With FLEX coils, the localization is easier, but the SNR at the stimulation site tends to be poorer due to the increased distance and artifacts introduced by the presence of the TMS coil. Recently, a different approach has been suggested, where a thin RF coil is mounted underneath the TMS coil thereby avoiding signal loss at the stimulation site (16, 46). With this thin surface coil array, regions further away from the coil array show lower SNR. However, it can be compensated by using additional coil arrays to cover other regions of the brain (47). The technical feasibility of combining TMS and fMRI coils in a single element has been established (48).

As for the TMS coil positioning system, Bohning et al. (49) suggested a holder that can be used close to an MR scanner and enables a manual positioning of the TMS coil based on the anatomical scan. Moisa et al. (50) developed a new TMS positioning system to reduce the set up time and improve TMS coil positioning to accommodate subject movement. Other research groups attempted to make their own TMS fixing system (43, 45, 51, 52). Nowadays, the manufacturers of MR-compatible TMS coils develop their own coil positioning systems in collaboration with researchers (50). Therefore, this is no longer a major concern for those starting to establish a concurrent TMS-fMRI system.

## Overview of Previous Concurrent TMS-fMRI Studies

The literature search was conducted initially on April 23rd, 2020, followed by another search on April 16th, 2021, to cover more recently published studies. Both searches were conducted following the same method except that the time frame was specified for the second search. The search was performed using PubMed scientific database as well as MEDLINE and Embase via Ovid. On both databases, the search keywords were as follows: (“transcranial magnetic stimulation” OR “TMS” OR “rTMS”) AND (“functional magnetic resonance imag^*^” OR “functional MRI” OR “fMRI” OR “functional connectivity” OR “fcMRI” OR “resting-state” OR “resting state” OR “rsMRI” OR “rsfMRI”). After deduplication, we identified 4,158 articles from the initial search and 715 articles from the second search. Through the screening and eligibility checks, we identified 73 and 5 concurrent TMS-fMRI articles with human subjects (sum of 78 articles) from the initial and second search, respectively (excluding one case study). This section focuses on providing an overview of the previous studies. The stimulation intensity and frequency (in the case of rTMS composed of more than 10 pulses per train) are indicated in the brackets. However, the parameters are very heterogeneous. For further detail of the study setup, it is recommended to look up the original publications.

### Concurrent TMS-fMRI Studies With Healthy Subjects

#### Overview of Motor Cortex Studies

##### Does TMS Induce a BOLD Effect?

Motor cortex is the most often studied brain region because the TMS response is easily assessable as a movement of a body part, such as finger twitches. The first-ever interleaved TMS-fMRI study was published in 1998 which showed a statistically significant BOLD signal increase in the motor cortex (0.83 Hz, 110%MT) (17). A later follow-up study showed that TMS over the motor cortex at 120%MT induces activations in the contralateral motor cortex and the auditory cortex, with the latter caused by the noise from TMS discharge (53). From the same group, it was demonstrated that higher TMS intensity (1 Hz, 110%MT) caused higher BOLD response amplitudes compared to low-intensity TMS (1 Hz, 80%MT) (18).

For rTMS, a linear relationship between the number of stimuli and the BOLD responses was reported for train lengths of up to 24 pulses (1 Hz, 120%MT) using a simple impulse-response model (54). Furthermore, it was shown that thumb movement induced by suprathreshold rTMS (1 Hz, 110%MT) over the motor cortex generates a similar BOLD signal pattern as if the thumb was moved volitionally (55, 56), and this result was confirmed for high-frequency rTMS (10 Hz, 110%MT) as well (57). Early studies on subthreshold rTMS [3–4 Hz, 90% active MT (AMT)] in the motor cortex did not find motor cortex activity (58, 59). Using a modified RF coil setup with increased sensitivity at the cortical target, a recent study confirmed that subthreshold stimulation (1 Hz, 80, 90%AMT) does not yield statistically significant increases in BOLD responses at the primary motor cortex (M1) target site (46). Applying rTMS to adjacent cortical areas of the M1 hand area (where no motor response is provoked) showed no consistent signal changes under the stimulated area (4 Hz, 150%AMT) (60). Therefore, it can be concluded that BOLD signal changes observed in the M1 hand area is the re-afferent somatosensory feedback of TMS-evoked movements, rather than any direct effects induced by the stimulation itself (41, 58–60). In fact, Denslow et al. (61) reported no significant difference between 100% MT TMS- and volition-induced effects (1 Hz). Interestingly, however, the authors showed qualitative differences in the BOLD signal time courses between stimulation and volition trials. Shitara et al. (41) conducted a more detailed examination of the time courses and spatial distribution of sTMS-induced fMRI signal changes and reported that neither the BOLD activity time courses nor spatial distributions were distinguishable between TMS and voluntary hand movements. However, the undershoot of the usual hemodynamic response function (HRF) was not observed with TMS-induced activities, except at the direct area (directly under the TMS coil) when TMS was applied with subthreshold intensity (randomized frequency, 90%AMT,). The TMS-induced activity was more deeply investigated by Shitara et al. (42) by subtracting the muscle twitch signal [by subtracting median nerve stimulation [MNS] induced sensory-related signal from MNS-induced muscle twitch signal] from the suprathreshold M1 TMS signal [randomized frequency, 120% of resting MT (RMT)]. As a result, a significant effect of TMS was still observed in M1, which suggests that TMS-evoked neuronal activations are not only sensory re-afferent related.

##### The Intensity and State Dependency of TMS-Induced BOLD Signals

The intensity of the BOLD signal depends on the intensity of the stimulation (46, 58, 59). A deeper investigation into this relationship revealed that BOLD intensity increases approximately linearly with subthreshold TMS intensity but non-linearly with suprathreshold intensity [0.15 Hz, 30–110% of maximum output (MO)] (62). Additionally, remote effects were found both with sub- and suprathreshold intensities, while direct effects at the area under the coil are observed only with suprathreshold intensity (58, 62). Shitara et al. (41) showed that the HRF lacks the typical undershoot after the signal peak at both direct and remote areas with suprathreshold single-pulse stimulation (120%RMT), but with subthreshold stimulation (90%AMT), the lack of undershooting was observed only at remote areas. With the subthreshold stimulation, the standard form of HRF was observed, but only at the direct area.

Moreover, the fMRI signal is not only stimulation intensity-dependent but also state-dependent. When comparing the neuronal activity induced by TMS at the dorsal premotor cortex (PMd) and M1, the activity change was state-dependent between the hand gripping task and resting state. During the hand gripping task, TMS (5 pulses at 11 Hz, 110%RMT) increased hemodynamic response in PMd and M1, but during the rest period, it decreased activity in the same area (63). When 1 Hz M1 rTMS is applied (100, 120%RMT), Jung et al. (64) reported that deductions in motor network activity are state-dependent too; rTMS decreases activity in motor networks during hand gripping, but it decreases even further during resting state. Furthermore, activations in the networks related to bodily self-consciousness were higher during resting state compared to task conditions.

##### The Network Effect of Motor Cortex TMS

TMS is known to produce a network effect—through the stimulated area, the effect of TMS can be observed at connected brain regions (65). This remote effect is observed with concurrent TMS-fMRI settings as well, which means that the propagation occurs immediately after the stimulation (61, 62, 66, 67). When TMS is given at the pre-motor cortex, the network effect is present with a subthreshold stimulation as well (3 Hz, 90%AMT), though the effect is smaller than with suprathreshold stimulation (3 Hz, 110%RMT) (66). Furthermore, the remote effect is present with both cortico-cortical and cortico-subcortical connections (66). Therefore, motor cortex stimulation can be administered to target deeper structures. For example, Hodkinson et al. (67) showed that TMS to M1 region (1 Hz, 100%RMT) modulates the connectivity between M1 and medial and lateral pain systems, which includes the insular cortex, anterior cingulate cortex (ACC), and parietal operculum cortex. This study presents credence to use M1 TMS for chronic pain patients.

##### Overview of Prefrontal Cortex Studies

Compared to the primary motor cortex, the effects of TMS over the prefrontal cortex are more difficult to observe. Despite this challenge, several researchers have targeted the prefrontal cortex with TMS to reveal the underlying mechanisms of neuronal modulation.

##### Intensity Dependency and Spatial Relationship

Studying prefrontal TMS with fMRI started by investigating the spatial relationship of prefrontal TMS with various intensities (68). They reported that 1 Hz rTMS to the prefrontal lobe at an intensity of 80%MT induces no significant activation except for the auditory cortex. At 100%MT stimulation, contralateral prefrontal activation was observed, and with 120%MT stimulation, bilateral prefrontal activation was observed with the contralateral side showing higher activation levels. These results were reported during the time when the motor cortex was heavily investigated and were in line with the result from the motor cortex stimulation, which showed BOLD signal reduction at the directly stimulated site (17). Twenty years later, Dowdle et al. (32) showed that sTMS to the dorsolateral prefrontal cortex (DLPFC) induces increased neuronal activity in the middle frontal gyri, insula, thalamus, and anterior cingulate cortex (ACC) with both active and sham (3 cm foam under TMS coil) stimulation (randomized frequency, 90–120%RMT). However, BOLD signal increases with active stimulation were greater in the ACC, caudate, and thalamus compared to sham control. Vink et al. (40) showed that sTMS to the DLPFC induces elevated activity in the prefrontal cortex, premotor cortex, primary somatosensory cortex, and subgenual ACC (sgACC), but not in the thalamus and insula (randomized frequency, 115%RMT). Furthermore, when sTMS was applied to the DLPFC, no linear relationship was observed between TMS and BOLD signal intensity (randomized frequency, 90–120%RMT) (32).

##### Prefrontal TMS-fMRI and Memory Function

Prefrontal regions, especially the DLPFC, are known to contribute to working memory (WM) function (69, 70). When TMS is applied to the DLPFC during memory encoding, it interferes with this function due to the virtual lesion effect (71). Feredoes et al. (72) used concurrent TMS-fMRI to show that DLPFC-TMS increases the activity in WM-related regions, such as the fusiform face area (FFA), which is related to face recognition, and the parahippocampal place area (PPA), related to environmental scene recognition (3 pulses at 11 Hz, 110%RMT). This effect, however, was only present when a distractor was present and was observed only in the region where current stimuli are represented (e.g., the effect is present in FFA only when a face is shown as a target and a house is shown as a distractor). This result provides valuable causal evidence that the DLPFC controls the stimuli-filtering function of the posterior area, which is consistent with previous studies (73–75).

In another TMS-fMRI study targeting semantic memory, Hawco et al. (76) showed that 10 Hz excitatory DLPFC-rTMS effects differ depending on the TMS onset timing (200, 600, or 1,000 ms post-stimulus) (3 pulses at 10 Hz, 100%RMT). These differences were observed at regions that are involved in higher-level cognitive processing (lateral frontal and anterior cingulate cortices) and semantic information processing (medial frontal and mid-temporal cortices), as well as at the visual cortex. This study provides another line of causal evidence that the DLPFC interacts with other WM process-related regions to control semantic memory encoding.

##### The Network Effect of Prefrontal TMS

Prefrontal TMS-fMRI studies also showed network effects. Hanlon et al. (20) reported that prefrontal sTMS (DLPFC and ventromedial PFC) activates the frontostriatal network, specifically in the prefrontal cortex, striatum, and thalamus (0.1 Hz, 100%RMT). This study showed that prefrontal TMS can also induce both cortico-cortical and cortico-subcortical network effects, as was the case for motor cortex stimulation (61, 62, 66). Thus, this network effect can be used to target a deeper structure. Oathes et al. (77) used a resting-state fMRI-guided sTMS system (randomized frequency, 120%RMT) to target an individual frontal area that is functionally connected with sgACC and amygdala. This study demonstrated that individually targeted TMS can modulate the sgACC distributed brain network, as well as the activity in the amygdala itself.

The prefrontal cortex has main nodes in three relatively well-studied networks that are known to be involved in higher cognitive functions: the central executive network (CEN) and the salience network (SN), which are less activated during the resting state (78, 79), and the default-mode network (DMN), which is reduced in activity whenever subjects attend to a specific task (80).

In a more recent study, the modulatory mechanisms between these networks were examined (51). It was shown that facilitatory sTMS (single pulses at 0.4 Hz, 120%RMT) to a CEN node (the posterior middle frontal gyrus) elicits negative connectivity between the DMN and CEN, and between the DMN and SN. On the other hand, inhibitory rTMS induced upregulation of DMN activity, though this effect was observed with an offline experiment where EPI was acquired immediately after 20 min of 1 Hz rTMS (120%RMT). Nevertheless, these results support the causal relationship of CEN in regulating the DMN activity, as has been suggested previously (79). Hawco et al. (81) showed that when the DLPFC has a stronger interaction with the SN, the correlation between intrinsic resting connectivity and TMS-induced changes in neuronal activity become stronger (randomized frequency, 100%RMT). From these applications, it may be concluded that concurrent TMS-fMRI is not only useful for investigating the direct effect of TMS in a certain functional network but also for examining the relationship between different functional networks.

#### Other Studies Within the Frontal Lobe

##### Frontal Eye Field

Frontal eye fields (FEF) are involved in controlling saccadic eye movement which plays an important role in visual attention and visuomotor control (82). Concurrent TMS-fMRI studies have revealed a top-down effect of the FEF on the modulation of the visual cortex. sTMS to the *right* FEF induces BOLD signal decreases in the central visual representation field, as well as BOLD signal increases in the peripheral field in V1–V4 (note that TMS to the *left* FEF induces effects only for central visual representation) (5 pulses at 10 Hz, 40–85%MO) (45). Ruff et al. (45) observed that FEF modulate activity in the retinotopic visual cortex, regardless of the presence of visual stimuli. When visual stimuli are present, right FEF-TMS elicits feature-specific effects; when attending to moving dots, the visual motion area shows an increase in cortical activity, whereas when attention is toward a face, the increase is observed in the fusiform face area (FFA) (3 pulses at 11 Hz, 110%RMT) (83). These findings support the idea that visual saccade attention is modulated via FEF feature-based functions.

#### Overview of Parietal Lobe Studies

The parietal lobe contributes to a wide range of complex functions, such as visuospatial attention, sensory processing, body awareness, language-related functions (such as writing, recognition, and naming of objects), and arithmetic processing. TMS to the parietal lobe can facilitate or inhibit these functions, and researchers have attempted to understand the mechanisms underlying these modulations. Furthermore, parietal stimulation also shows a network effect.

##### Parietal TMS-fMRI and Visuospatial Function

The first parietal concurrent TMS-fMRI study was conducted by Sack et al. (84) exploring visuospatial judgments. Sack and his colleagues showed that right, but not left, parietal TMS (randomized frequency, 100%MO) interferes with visuospatial processing by modulating the right-hemispheric frontoparietal network, indicating that the parietal cortex interacts with frontal areas to regulate visuospatial attention. Consequently, Ricci et al. (36) confirmed with three subjects that right suprathreshold posterior parietal cortex (PPC) sTMS (115%RMT) induces rightward bias with the line bisection task due to neglect-like behavior produced in the left visual hemifield, and this effect was observed in frontoparietal regions. Additionally, Blankenburg et al. (44) found that the effect of the PPC stimulation intensity, which was observed in the occipital visual cortex during a visuospatial attention task, depends on the visually attended side; when visual attention is toward the contralateral side, a larger difference in BOLD signal between high (75%MO) and low (35%MO) TMS intensity condition was observed compared to ipsilateral visual attention (5 pulses at 10 Hz). Regarding visuospatial attention, the neuronal activity of the right angular gyrus (AG) is considered to play a crucial role when reorientation of visuospatial attention is required, for example, by receiving a wrong cue. The right AG TMS (three pulses at 11 Hz, 120%RMT) elicited a facilitatory effect when the target was on the right hemifield following the invalid cue, meaning that right AG TMS facilitates rightward spatial reorientation. The neuronal effect was observed in the left AG and left visual area, suggesting that there is interhemispheric interaction between the right AG and remote connected areas in the left hemisphere. Moreover, this study showed that the right AG also influences the neuronal activity of the visual cortex, in addition to the right PPC (27).

##### Parietal TMS-fMRI With Visual Stimuli

With regards to the interaction with the visual cortex, the intra-parietal sulcus (IPS) is a region that has been relatively well-investigated with concurrent TMS-fMRI. IPS is known to contribute to visual-motor coordination (such as saccade preparation and grasping objects) (85). IPS TMS increases activation in the parietal cortex during resting state without any visual stimuli (10 Hz, 69%MO) (86). However, when visual stimuli are present, IPS TMS increases cortical activation in the cuneus, and this activation at the cuneus decreases when no visual stimuli are shown (four pulses at 10 Hz, 66%MO) (87). For moving visual stimuli, Ruff et al. (28) showed that TMS over the right IPS interacts with the occipital visual cortex depending on the visual context (five pulses at 9 Hz). With higher intensity IPS TMS (tested with 40–85%MO), the BOLD signal increase was observed in visual motion areas but only when stimuli were present and moving. On the other hand, when stimuli were absent, the effect was observed in V1–V4 visual retinotopic areas. Furthermore, it was confirmed that TMS over the right IPS is more effective than over the left IPS; right TMS induced stronger BOLD signal modulation in the visual cortex but left TMS did not induce any significant difference (45) (five pulses at 9 Hz, tested with 40–85%MO).

As for low-salience visual stimuli, IPS TMS (four pulses at 10 Hz, 69%MO) modulates neuronal activation. In the no-TMS condition, detecting weak visual stimuli showed activation increases in the anterior insula, which is a crucial node of the ventral attentional network for salience detection, and decreases in the ventral visual area. However, with IPS TMS, the activation increased in the right temporoparietal junction (TPJ), which is also a node of the ventral attentional network, and decreased in the right fusiform area (86). This IPS function of low salience stimuli detection is useful for the brain to decide which visual information is relevant from the sensorially noisy environment. IPS TMS did not improve accuracy in finding low saliency stimuli but did quicken response times following the error trials (88). Furthermore, IPS TMS attenuated activity increases in the left middle and superior frontal gyri, which was only observed in the missed visual stimuli case but not when correctly seen (four pulses at 10 Hz, 69%MO) (88). Hence, the IPS is considered to be involved in the post-decisional process by reflecting the decision accuracy and confidence (88).

##### Parietal TMS-fMRI and Sensorimotor Integration

Another important function of the parietal cortex is sensorimotor integration, such as assisting hand movements in line with the goal orientation (89). de Vries et al. (90) found that impaired function of the superior parietal cortex, which is related to the proprioceptive adjustment of spatial movement control (91), leads to an increase in BOLD signal in remote areas. Suprathreshold TMS at the frequency of 1 Hz (115%MT) was given over the left superior parietal cortex prior to hand movement execution. The result showed increased activity in bilateral prefrontal, right temporo-parietal, and left posterior parietal cortices. Therefore, these remote areas may compensate for any functional impairments of the superior parietal cortex (90).

##### Parietal TMS-fMRI With Somatosensory Function

TMS over the right parietal cortex was conducted to investigate the neural association of somatosensory function. Blankenburg et al. (43) demonstrated that TMS to the right parietal cortex (five pulses at 10 Hz, 110%RMT) modulates BOLD signals in the left primary somatosensory cortex (SI), but that this depends on whether the somatosensory input is present or not; with somatosensory input (MNS to the right wrist in this case), the neural activity in SI increases, whereas it decreases without somatosensory input. A similar effect was observed in the thalamus with the region of interest (ROI) analysis. This study suggests that the right parietal cortex is involved in the somatosensory processing in the left SI.

##### The Network Effect of Parietal Stimulation

Targeting the hippocampus enables us to investigate its role in episodic memory. Hermiller et al. (92) applied TBS to the lateral parietal cortex, which is a part of the hippocampal network, and demonstrated that the left hippocampus shows increased neuronal activity during scene encoding and the subsequent recollection was significantly better when performed after the TBS (80%RMT). This study suggests the ability of TBS to influence hippocampal memory function.

Overall, these parietal concurrent TMS-fMRI studies suggest heavy interaction between the parietal cortex and occipital visual cortex. It is considered that high-level adaptive behavior is processed as an integration of bottom-up sensory inputs and top-down control signals to adjust the current action to meet the task goal. In the case of visual control, the parietal cortex supports the visual function of the occipital lobe to fine-tune its visual actions. Moreover, these concurrent TMS-fMRI studies show that right hemispheric structures induce stronger effects in the visual cortex compared to the left.

#### Overview of Occipital Lobe Studies

##### Occipital TMS-fMRI and Phosphene

The occipital lobe is the central system of visual processing. TMS to the primary visual cortex in the occipital lobe is known to induce phosphenes, i.e., the perception of transient light. This phosphene threshold (PT) is another method to determine the TMS intensity. The neural correlates of the TMS-induced phosphene have been mostly studied with EEG (93–95), but also some have investigated this phenomenon with fMRI. Since phosphenes are subjective, it is difficult to find its neuronal correlates with small sample sizes. In fact, de Graaf et al. (96) reported no meaningful observation in cortical activity modulation associated with phosphenes, but this study included only four subjects (randomized frequency, 80–120% phosphene threshold; PT). However, consistent with previous research (97), Caparelli et al. (98) employed a concurrent TMS-fMRI paradigm and reported that considerable differences in activity are observed in the visual network between those who perceive phosphenes and those who do not (0.25 Hz, 100%PT). These studies imply that a functional distinction within visual networks separates subjects who experience phosphenes from those who do not, and this is possibly the origin of phosphene generation (98).

### Concurrent TMS-fMRI Studies With Clinical Subjects

#### Psychiatric Disorders

##### DLPFC TMS-fMRI With Depression

In recent years, the number of concurrent TMS-fMRI studies with psychiatric patients has increased. With MDD patients, DLPFC stimulation has induced elevated local and global network effects, both directly under the coil and in connected subcortical regions, such as the thalamus, putamen, and insula (1 Hz, 100%MT) (99), which is in line with other studies involving healthy subjects (32, 68). Following these studies, Eshel et al. (100) conducted a DLPFC TMS-fMRI study with MDD and healthy controls, specifically targeting the DLPFC node of the frontoparietal control network. This study showed that DLPFC stimulation activates the right DLPFC in patients with MDD, but not in healthy controls. Eshel and colleagues also report that DLPFC stimulation inhibits amygdala activity in healthy controls, but not in patients with MDD (0.4 Hz, 120%RMT) (100).

##### DLPFC TMS-fMRI With PTSD

The efficacy of DLPFC stimulation for post-traumatic stress disorder (PTSD) is another avenue for TMS research. A concurrent TMS-fMRI study reported that right DLPFC stimulation to PTSD patients induces an inhibitory effect in the left amygdala. Furthermore, a positive correlation was reported between the degree of inhibition and the outcome of the typical exposure psychotherapy (101). Another study investigated PTSD patients with frontopolar stimulation and found that the ventromedial prefrontal cortex, which is related to emotion regulation and is usually downregulated via the frontopolar cortex, can be deactivated with frontopolar TMS as well (102) (both studies: 0.4 Hz, 120%RMT).

##### Prefrontal TMS-fMRI With Substance Abuse

As for cocaine users, left DLPFC stimulation did not evoke a significant difference in BOLD signal compared to healthy controls (33). However, TMS over the medial PFC led to lower ventral striatal activation in cocaine users compared to healthy controls (0.08 Hz, 110%RMT) (33). rTMS applied to the frontal pole induces BOLD responses at the striatum and salience network in cocaine users (randomized frequency, 100%RMT) (103). Furthermore, Hanlon et al. (104) investigated the effects of TMS on BOLD signals before and after frontal pole continuous theta-burst stimulation (cTBS) with cocaine and alcohol users. Compared to the pre-cTBS fMRI scan, the post-cTBS scan revealed inhibition at the orbitofrontal cortex as well as at regions that are related to salience regulation, which are known to be activated by drug usage (0.1 Hz, 110%RMT).

##### Prefrontal TMS-fMRI With Schizophrenia

Guller et al. (105) showed that precentral gyrus TMS administered to schizophrenia patients evokes decreased BOLD responses in the thalamus and medial superior frontal cortex compared to healthy controls. Additionally, reduced connectivity between the thalamus and superior frontal gyrus, as well as between the thalamus and insula, was observed in this study (single pulse, 110%MR). Webler et al. (106) stimulated the left frontal cortex (Brodmann area 9; BA9) of schizophrenia patients with 10 Hz triplet pulses (80–120%RMT; cortical distance adjusted). This study reported stronger activity in left BA9 and neighboring BA46 compared to healthy controls. Furthermore, disrupted interhemispheric functional connectivity between left and right BA9 was demonstrated with schizophrenia patients.

#### Neurological Disorders

With neurological disorders, Bestmann et al. (26) conducted a concurrent TMS-fMRI study with post-stroke patients. The study showed a stronger BOLD signal effect in posterior regions of the ipsilesional sensorimotor cortex induced by contralesional dorsal premotor TMS during handgrip, which is associated with more severe clinical and neurophysiological post-stroke impairment (five pulses at 11 Hz, 110%RMT). With cervical dystonia patients, 1 Hz rTMS (115%RMT) to the left superior parietal cortex led to significantly less activation in the right angular gyrus compared to healthy controls (107).

As for epilepsy, no concurrent TMS-fMRI studies have been conducted yet. However, a series of concurrent TMS-fMRI studies from Li and colleagues investigated the psychopharmacological effect of two anticonvulsant drugs, lamotrigine and valproic acid, with healthy subjects. After lamotrigine intake, TMS to the motor cortex inhibited cortical activity overall compared to placebo (no detail of frequency, 100, 120%RMT). However, prefrontal TMS promoted activity in the orbitofrontal cortex and hippocampus, which indicates an effect of lamotrigine in corticolimbic circuits (108). With valproic acid, an inhibitory effect was observed with motor cortex TMS (five pulses at 1 Hz, 100,120%RMT). However, the facilitatory effect of prefrontal TMS was not observed (35). Further investigation indicated that both lamotrigine and valproic acid have an inhibitory effect in the connectivity between the M1 and pre-motor cortex, as well as between M1 and the supplementary motor area, after motor cortex TMS (five pulses at 1 Hz, 100,120%RMT). Moreover, lamotrigine, but not valproic acid, has a facilitatory effect in the network between the left DLPFC and ACC (34).

## Limitation and Future Direction

Although concurrent TMS-fMRI has helped us to understand the neural correlates of TMS in an unprecedented manner, there are still some technical limitations that should be considered. One of them is the temporal resolution of concurrent TMS-fMRI. For example, with the inter-volume protocol, where the TMS is applied during the gap time between EPI volumes, the stimulation protocol is limited to the length of the repetition time (TR) of the fMRI sequence, which is typically around 2 s. With the inter-slice protocols, where TMS pulses are interleaved with EPI slices, continuous image acquisition is possible with frequencies up to 10 Hz (109). However, a precise interleaving of EPI sequences and timing of TMS pulses remains complex.

Other challenges that need to be addressed in concurrent TMS-fMRI are (1) restricted spatial selection of the stimulation target due to spatial constraints within the MR coils, (2) subject movements that increase the distance between the TMS coil and stimulation target, (3) sham conditions. To overcome spatial constraints, recent approaches sometimes use flex-coils that can be dynamically placed around an area of interest (40), or a thin RF receiver coil on which the TMS coil can be mounted (16, 46).

Minimizing the subject's motion during a TMS-fMRI session is critical to ensure. Head movement can affect not only scanning quality but also TMS efficacy by accidentally increasing the coil-head distance. The spatial flexibility of coil localization and motion minimization are often inversely related, i.e., the birdcage MR coil is less flexible with TMS coil localization but easier to fixate the head [pictures of the actual setup can be found in Bestmann et al. (59) and Hodkinson et al. (67)]. When flexible MR coils are used, the head fixation becomes difficult as there is no frame where sponges can be inserted. It is recommended to create a wall around the head to minimize the head movement [for example, Figure 1B uses a deflatable pillow. For another example, see Vink et al. (40)]. Regarding the motion tracking in the scanner, it has recently been proposed to extract subject motion information from alignment parameters obtained from EPIs (110). However, this method is limited by spatial (about 1 mm) and temporal resolution (typically 1–2 s, defined by the TR). To control the motion effect, online visual feedback procedures and online dose adjustment have also been proposed (111). The idea is to track the head and coil locations using a neuro-navigation camera throughout the TMS-fMRI sessions (Figures 1C,E). It requires a system that is both MR-compatible and detectable within the narrow scanner bore. Further development of motion minimization and tracking systems are required.

Regarding sham conditions, the recent approach is to increase the distance between the coil and the scalp by placing a plastic block between the TMS coil and the scalp, thereby avoiding effective stimulation (32, 40, 112), or between the MR receiver coil and the TMS stimulation coil when a 7-channel concurrent TMS-fMRI coil array is used (113). Tik et al. (113) showed that this approach resulted in an activation increase in somatosensory areas during sham and verum stimulation, with only the latter resulting in an increase in DLPFC activity during verum stimulation.

As the number of studies increases, the technical aspects of concurrent TMS-fMRI have dramatically improved over the past decades. However, setting up the concurrent TMS-fMRI environment still requires a considerable amount of time and knowledge. Due to its complexity, it often requires the study participants to stay still for a long time, which makes it even more difficult to employ patients. As shown in this review, most studies have been conducted with healthy subjects, while clinical populations are underrepresented. To lower the barrier of the TMS-fMRI system implementation for clinical researchers, knowledge should be pooled and shared for technical solutions. A systematic review of concurrent TMS-fMRI should be also referred to when a new study design is developed (114).

Furthermore, previous concurrent TMS-fMRI studies mainly investigated the effect of the motor cortex, while other brain regions are still underrepresented—especially posterior regions, such as the occipital cortex, where it is difficult to stimulate subjects in the supine position on the scanner bed. To date, ~80% of concurrent TMS-fMRI studies have investigated the motor cortex. With the help of novel developments in TMS-fMRI hardware, future studies may extend TMS-fMRI research to other areas of the brain.

Last but not least, recent technological improvements showed the feasibility of concurrent TMS-EEG-fMRI (15, 115, 116). The combination of EEG and fMRI covers both temporal and spatial resolution. Therefore, concurrent TMS-EEG-fMRI allows for the investigation of TMS effects in a widely distributed network with higher accuracy over time.

Concurrent TMS-fMRI will contribute to increased biological validity. TMS can modulate neuronal activity at different cortical areas. However, the mechanism of cortical excitation and inhibition is not completely understood yet. Moreover, the relationship between neuronal modulation and behavioral consequences remains a black box. Clinical protocols can be better individualized to achieve a higher treatment efficacy when it becomes clearer how neuronal networks, hub regions, and read-outs such as inter- and intra-hemispheric connections are interacting. We strongly encourage all TMS-fMRI researchers to collaborate and share knowledge and experiences of this extremely complicated but powerful technique. An effective way to achieve this is to share data and exchange knowledge through Open Science platforms, such as OSF (https://osf.io/), zenodo (https://zenodo.org/), or Gitlab (https://gitlab.com/gitlab-org/gitlab).

## Author Contributions

YM-T and MT gathered the knowledge and wrote the review. CW, FP, and DK supervised and provided the corrections and feedback for improvements. K-YC and AS supported to make the list of previous studies. ZW, CV, and LB supported the set-up of TMS-fMRI system. All authors contributed and approved the final manuscript.

## Funding

The study was done using the NICUM Siemens Prisma scanner supported by German Research Foundation (DFG; Deutsche Forschungsgemeinschaft) [grant number INST 86/1739-1 FUGG].

## Conflict of Interest

FP was a member of the European Scientific Advisory Board of Brainsway Inc., Jerusalem, Israel and has received speaker's honoraria from MagandMore GmbH and the neuroCare Group. His lab has received support with equipment from neuroConn GmbH, Ilmenau, Germany, MagandMore GmbH, and Brainsway Inc., Jerusalem, Israel. YM-T was a half-time employee of neuroCare Group and this work is a part of her Ph.D. program at Munich Medical Research School. The remaining authors declare that the research was conducted in the absence of any commercial or financial relationships that could be construed as a potential conflict of interest.

## Publisher's Note

All claims expressed in this article are solely those of the authors and do not necessarily represent those of their affiliated organizations, or those of the publisher, the editors and the reviewers. Any product that may be evaluated in this article, or claim that may be made by its manufacturer, is not guaranteed or endorsed by the publisher.
